# Protocol for estimating the impact of climate change on economic growth and inequality under climate policies

**DOI:** 10.1016/j.xpro.2023.102527

**Published:** 2023-08-25

**Authors:** Yun Tang, Hongbo Duan, Shi-Yun Yu

**Affiliations:** 1 School of Economics and Management, University of Chinese Academy of Sciences, Beijing 100190, China; 2 College of Oceanic and Atmospheric Sciences, Ocean University of China, Qingdao 266100, China

**Keywords:** Computer Sciences, Environmental Sciences, Earth Sciences

## Abstract

The impact of climate change on economic inequality has attracted increasing attention from both government and academia. Here, we present a protocol for estimating both the impact of climate change on economic growth and economic growth inequality under multiple climate policies. We describe steps for constructing an uncertainty analysis framework, collecting and pre-processing data, and estimating the climate-economic response. We then detail procedures of predicting climate policy impact and calculating inter-country economic growth inequality.

For complete details on the use and execution of this protocol, please refer to Tang et al. (2023).^1^

## Highlights

• Estimates the loss-response relationship between climate change and economic growth
• Construct an uncertainty framework for assessing climate policy under 68 scenarios
• Project the impact of climate policies on economic growth in different future periods
• Calculate inter-country economic growth inequality under climate policies

## Abstract (editor highlights)

Publisher’s note: Undertaking any experimental protocol requires adherence to local institutional guidelines for laboratory safety and ethics.

## Graphical abstract

**Figure:**
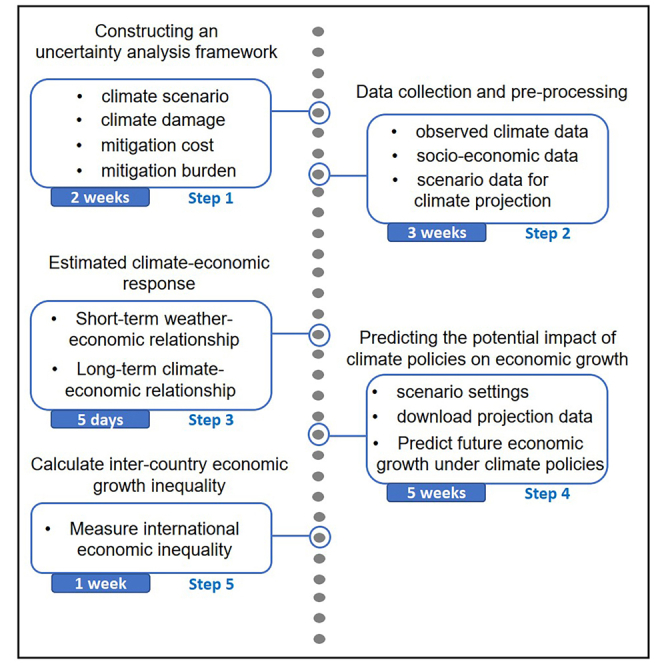

## Before you begin

As climate change becomes more severe, it is important to assess whether climate policies can synergistically mitigate climate change, promote economic growth, and improve economic inequality. The protocol given below describes the general design of the study, with specific steps on how to construct an uncertainty analysis framework for assessing climate policy. Based on this, the protocol calculates the impact of climate policies under multiple scenarios on future economic growth at the near-, medium-, and long-term scales. Furthermore, it measures the dynamic evolution of the growth of global economic inequality during the 21st century.

### Study design

**Figure 1:**
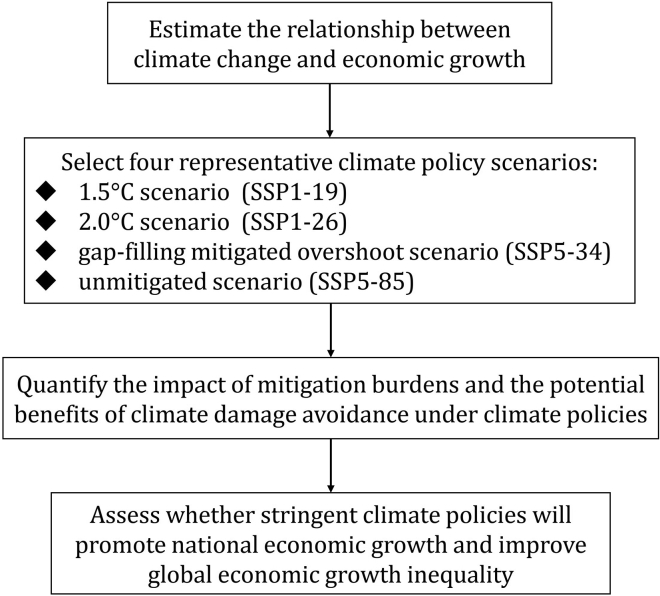
Analysis steps

This study first estimates the loss-response relationship between climate change and economic growth; Second, select four representative climate policy scenarios ranging from strict to lax, in order of 1.5°C scenario (SSP1-19), 2°C scenario (SSP1-26), gap-filling mitigated overshoot scenario (SSP5-34), and unmitigated scenario (SSP5-85); Third, simulate and predict the economic costs of climate policies and the economic benefits from damage avoidance; Finally, assess whether strict climate policies can synergistically mitigate climate change, promote economic growth, and improve economic inequality. Figure 1 summarizes the logical framework proposed in this paper.

Specifically, this study considers 1961–2017 as the baseline period and three alternative baseline periods (1971–2017, 1978–2017, 1991–2017) to estimate the loss-response relationship between climate change and economic growth. Then, two medium- and long-term projection periods, i.e., mid-century (2045–2055), and end-century (2090–2100) are defined to assess the potential impacts of 4 representative climate policies ranging from strict to lenient on future economic growth in 171 countries. Further, we select 18 representative countries to assess the economic impacts of climate policies at different time scales in the near (2030–2040), medium (2050–2060), and long term (2070–2080, 2090–2100). Finally, we measure the dynamic evolution of global economic growth inequality under 68 scenarios over the 21st century (2015–2100).

In this work, our computer configuration is Intel i9-13900K, RTX1660 Ti independent display card, 2 TB hard drive, 64 GB of memory.

For the processing of meteorological data, we used the NCAR Command Language (NCL, Version 6.6.2). The NCL is a free interpreted language designed specifically for scientific data processing and visualization, which is a product of the Computational & Information Systems Laboratory at the National Center for Atmospheric Research (NCAR) and sponsored by the National Science Foundation.

### Uncertainty analysis framework

**Timing: 2 weeks**

**Figure 2:**
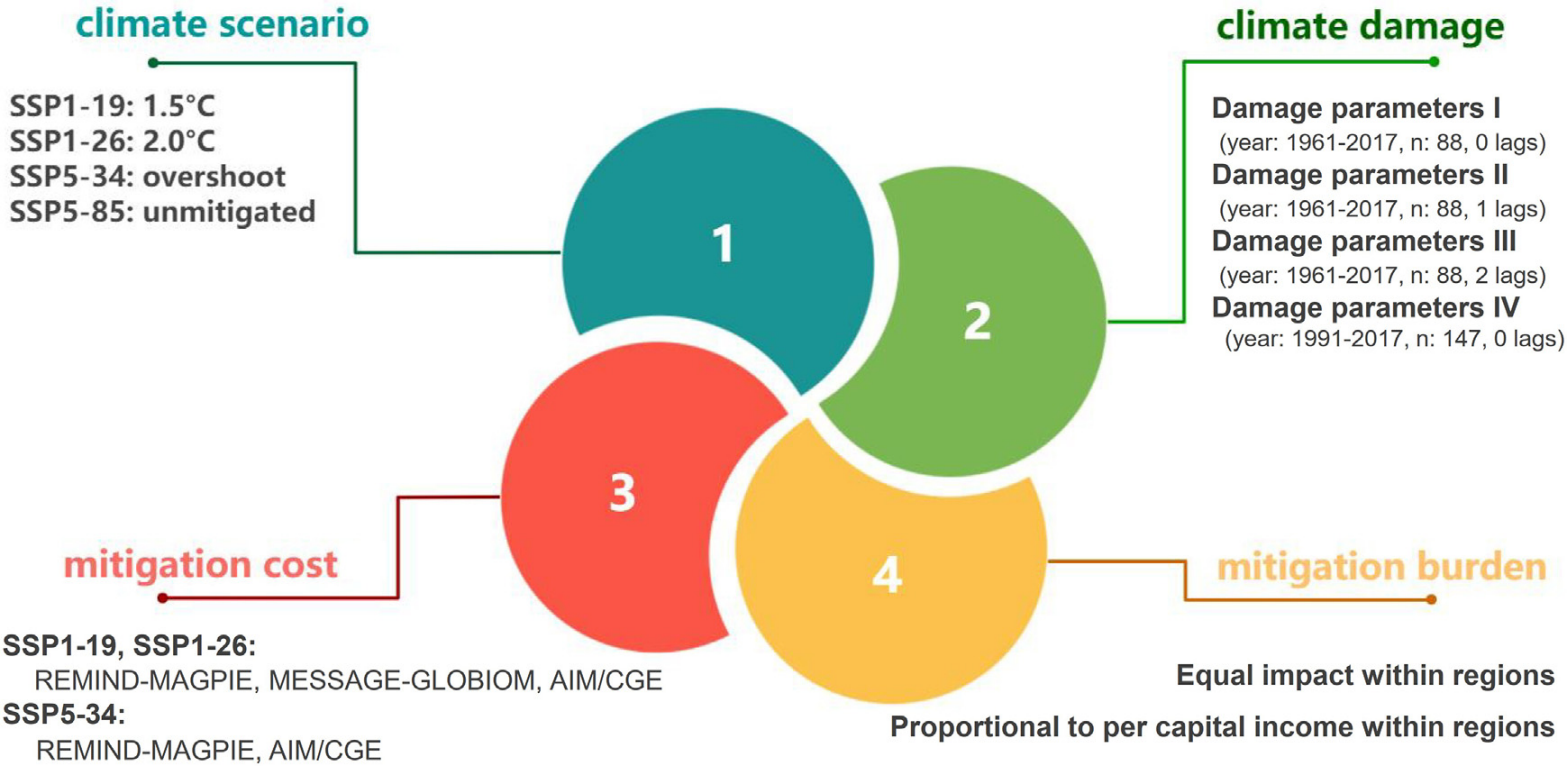
Uncertainty analysis framework

Aiming at assessing the long-term potential economic impacts of near-term climate policies under multiple scenarios, we construct an uncertainty analysis framework with four uncertainty dimensions: climate policy, climate damages, policy costs, and mitigation burdens. Figure 2 shows the uncertainty analysis framework proposed in this paper.

**Figure 3:**
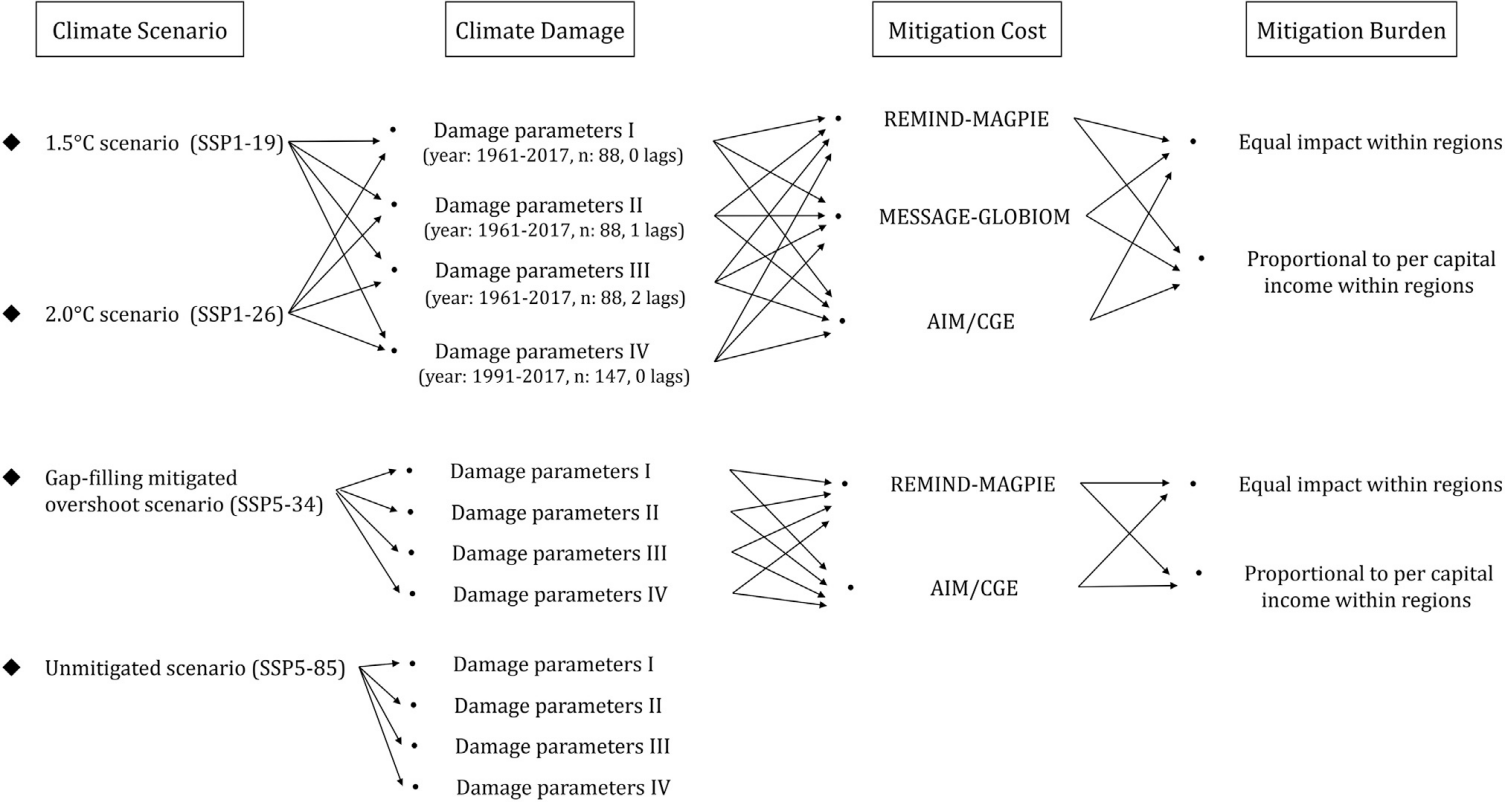
Scenario library

The first dimension is the **climate scenario**, which refers to four representative climate policy scenarios from strict to loose (SSP1-19, SSP1-26, SSP5-34, SSP5-85). It reflects the varying degrees of global average warming from low to high by the end of the 21st century (2100). The second dimension is **climate damage**, which is estimated from the loss-response relationship between climate change and economic growth. It captures the unequal impact of varying degrees of climate damage on economic growth. Third, the **mitigation cost** dimension involves calculating the mitigation costs of different regions using multiple integrated assessment models (IAMs) that include endogenous economic growth modules (AIM/GCE, REMIND-MAGPIE and MESSAGE-GLOBIUM), as they represent the effect of mitigation on growth.^2^ Last, the **mitigation burden** dimension is used to portray the heterogeneous impact of mitigation costs on economic growth in different countries within the same region. Here, we are inspired by the study of Taconet et al. (2020)^2^ and further postulate that the mitigation burden affects national economic growth in two scenarios based on two different principles of equity and efficiency. It reflects differential distributions of mitigation efforts (see Tang et al. (2023)^1^ for details). By combining different scenarios from each of these four dimensions one by one, we obtain 68 scenarios with different economic implications and form a scenario library to assess the long-term potential impacts of near-term climate policies, as shown in Figure 3.

### Data collection and pre-processing

**Timing: 3 weeks**

1. Observed climate data.
  a. The observed climate data are obtained from the database of the Climate Research Center at the University of Delaware. This database provides 0.5 degree grid monthly average temperature and total precipitation data for the global land area from 1900 to 2017 (Version 5.0),^3^ as interpolated from meteorological station data. Further, average monthly temperature and precipitation data over the period 1961 to 2017 to obtain annual averages.
    i. Calculates temperature and precipitation on a country-year scale weighted by **administrative area** within global land areas.
      ***Note:*** Administrative area is a geographical area within a country or region that is managed and controlled by the government. It is defined as land area at the national level and is used to weight climate variables to measure annual average temperature and total annual precipitation for each country.
    ii. Combined with the raster data of world population density provided by the National Aeronautics and Space Administration (NASA),^4^ we calculate the average temperature and precipitation on a country-year scale weighted by **population density** in the global land area.
2. Socio-economic data.
  a. Socioeconomic data comes from the World Development Indicators (WDI) database. To match socioeconomic data with observed climate data, this study takes annual socioeconomic data for the same time period, i.e., 1961 to 2017, 88 countries.
    i. The national socio-economic data and the population-weighted meteorological data at the country-year level are matched one to one precisely, according to the latitude and longitude information of the administrative regions in the world.
    ii. In the robustness testing section, to reduce the effect of sample selection bias on the estimation results of this study, the paper re-matches three balanced panel datasets that are shorter than the baseline period (1961 to 2017) but contain more sample countries, namely: (1)106 countries between 1971 and 2017; (2)111 countries between 1978 and 2017; (3) 147 countries between 1991 and 2017.

**Figure 4:**
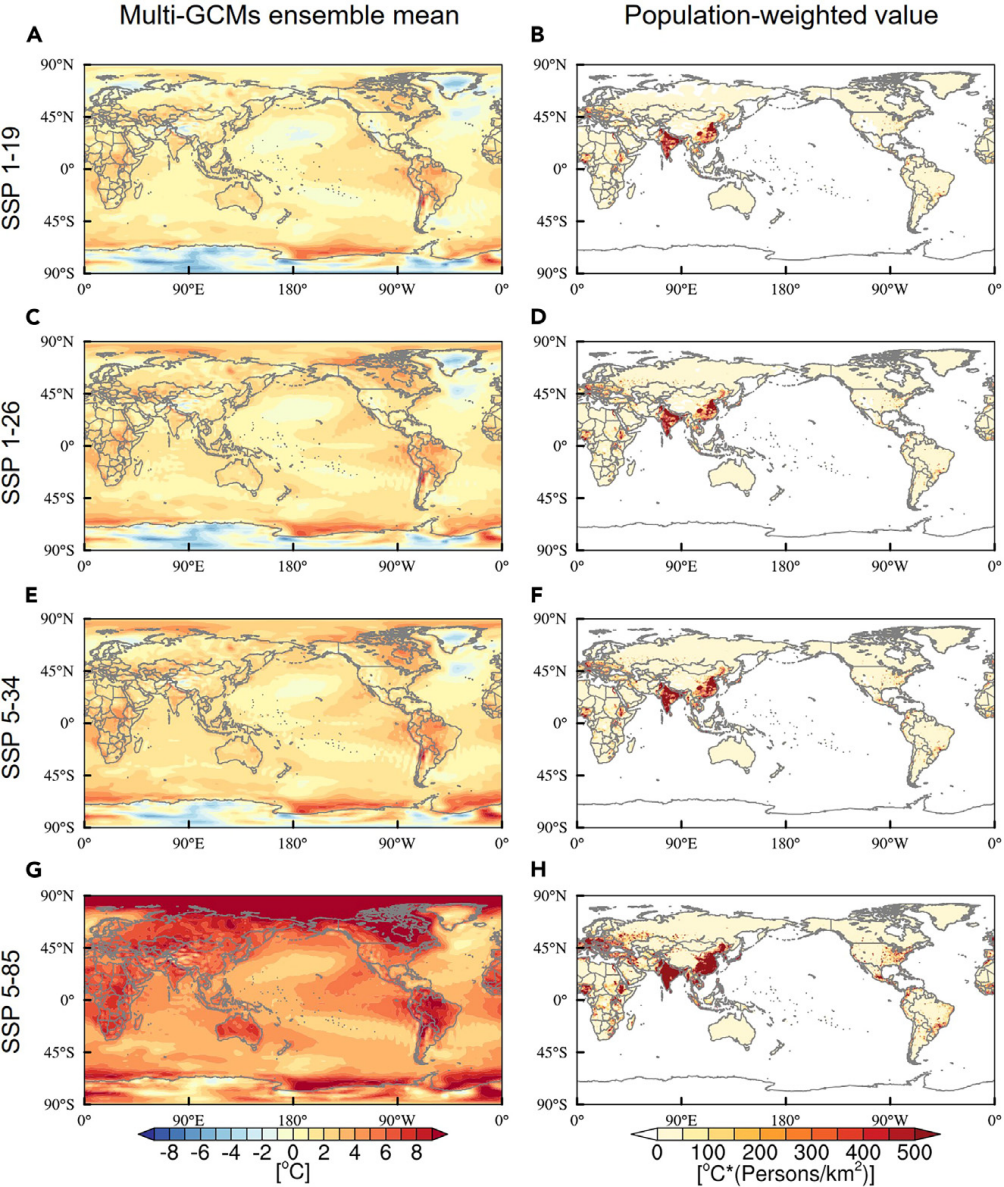
Scenario data for climate projections in 2100 (A–H) Note: (A), (C), (E), (G), Climate projections of multi-GCMs ensemble mean at 2100 for SSP1-19, SSP1-26, SSP5-34, SSP5-85 scenarios. (B), (D), (F), (H), Population-weighted climate projections of multi-GCMs ensemble mean for the corresponding scenario.
3. Scenario data for climate projection.
  a. Future temperature prediction data is provided by the Scenario Model Comparison Program (ScenarioMIP), a key subprogram of the Phase 6 of the Coupled Model Intercomparison Project (CMIP6). Specifically, CMIP6 has dozens of Global Climate Models (GCMs) that provide key data support for future SSP-RCP scenarios.
    i. To assess the economic impacts of representative climate policies ranging from strict to lenient, this study uses monthly surface temperatures for the future period (2015–2100) for the SSP1-19 (1.5°C), SSP1-26 (2.0°C), SSP5-34 (overshoot), and SSP5-85 (unmitigated) scenarios.
    ii. Future temperature projections are provided by ten available global climate models (GCMs), they are CanESM5, CNRM-ESM2-1, FGOALS-g3, GISS-E2-1-G, GISS-E2-1-H, MIROC-ES2L, IPSL-CM6A-LR, MIROC-ES2L, MRI-ESM2-0 and UKESM1-0-LL.
      ***Note:*** CMIP6 provides a large amount of model data run by many research institutions to study climate change. However, many institutions have only run basic experiments and have not run experiments under different future scenarios. In this paper, we select four experiments under representative SSP-RCP scenarios to describe different warming pathways, SSP1-19 (1.5°C), SSP1-26 (2°C), SSP5-34 (mitigated) and SSP5-85 (unmitigated). To ensure that the model results are more reliable, we need to pick the models that are present in all four scenarios. Considering the availability of four representative SSP-RCP scenarios, as well as securing the same realization number and initial conditions (variant level “r1i1”), only ten GCMs are available. Therefore, we have selected these 10 models for the study of this paper. We also look forward to more institutions running these four experiments in the future to provide more models data. Besides, in this study, we use the results of multi-GCMs ensemble mean to reduce the effect of simulation bias for a single model.
    iii. To simplify the analysis, this study averages the 10 GCM outputs for future temperature predictions.
    iv. In order to obtain the multi-model ensemble mean-based results, this study uses bi-linear interpolation method to reduce the 10 GCM outputs to a consistent 0.5 degree resolution. As shown in Figures 4A, 4C, 4E, and 4G.
    v. Consistent with the climate data pre-processing methods observed during historical periods, we perform population weighting on the multi-GCMs ensemble mean (as shown in Figures 4B, 4D, 4F, and 4H).

## Key resources table

**Table:** 

REAGENT or RESOURCE	SOURCE	IDENTIFIER
**Deposited data**		

Country-level GDP per capita growth	World Bank	https://data.worldbank.org.cn/
Projections of future economic growth	SSP database	https://tntcat.iiasa.ac.at/SspDb/
Observed climate data	University of Delaware	http://climate.geog.udel.edu/
World population density	NASA	https://doi.org/10.7927/H49C6VHW
Projections of climate data	CMIP6	https://esgf-node.llnl.gov/projects/cmip6/

**Software and algorithms**		

Stata	Stata	Stata 17
Arcgis	Esri	Arcgis 10.2
Matlab	MathWorks	R2020a

## Step-by-step method details

In this section, we first estimate the response relationship between climate change and economic growth. On this basis, the impact of future warming on economic growth under climate policy is projected. Finally, we measure the evolutionary trend of cross-country economic growth inequality between the 21st century under different climate policies and scenarios.

### Estimated climate-economic response

**Timing: 5 days**

This step distinguishes between short-term weather shocks and long-term climate change impacts on economic growth. The differentiated impacts of short-term weather fluctuations versus long-term climate change on economic growth are effectively identified by defining short-term weather variables as annual temperature and precipitation and their differentials, and long-term climate variables as the differentials of annual temperature and precipitation deviations from their historical norms (the past decades).

1. Short-term weather-economic relationship.
  a. Define climate variables.
    i. Annual averages: arithmetic mean of population-weighted temperature (precipitation) for 12 months per year
    ii. Differential forms: the magnitude of temperature (precipitation) change in year *t* compared to year *t-1.*
  b. Set forms of specifications, compare and select the best performing equation as the baseline model.
    i. Linear.^5^^,^^6^
    ii. Nonlinear.^7^^,^^8^
    iii. Difference.^9^
  c. Heterogeneity analysis.
    i. Poor country *vs*. Rich country.
    ii. Colonized country *vs*. Non-colonial country.
    iii. Hot country *vs*. Cold country.
      ***Note:*** (1) Poor/Rich country is defined as having below-median/above-median PPP-adjusted per capita GDP in the first year the country enters the datasets. (2) Colonized/Non-colonial country refers to a country that has/hasn’t experienced colonialism. (3) Hot/Cold country is defined as having above-median/below-median annual average temperature in the first year the country enters the datasets.
  d. Lagged effects: gradually incorporate 0th to 5th order lagged terms for temperature and precipitation variables into the baseline model.
  e. Robustness: changing periods, sample countries, and econometric methods.
    ***Note:*** In the specifications, this study includes the rate of temperature change ($\Delta {Tr}_{i,t}$), which reflects the speed of temperature change. The results indicate that for the same amount of warming ($\Delta T_{i,t}$), the faster the temperature rises, the slightly lower the economic growth. For example, rapid 2°C warming over 10 years has greater negative impact on economic growth than slow 2°C warming over 100 years.
2. Long-term climate-economic relationship.
  a. Define climate variables.
    ***Note:*** Climate change usually refers to changes in the average state of the climate that occur over a longer period of time.^10^^,^^11^ The larger the deviation, the greater the magnitude of climate change.
  b. Identify changes in long-term climate conditions.
    ***Note:*** Referring to Kahn et al. (2021),^12^ this study defines climate variables as the difference between annual temperature/precipitation and their moving averages over the past few decades.
  c. Define history norms.
    ***Note:*** Typically, climate norms are measured by the moving average of the past 30 years.^13^
  d. Robustness check.
    ***Note:*** Define climate variables as the difference between the annual temperature and precipitation variables deviating from their 20-, 40-, and 50-year’s moving averages. When using different numbers of decades as historical norms, we choose the most appropriate data set among the four balanced panels mentioned above to reduce sample loss. For example, when using the 20-year moving average as the historical norm, we choose the balanced panel dataset of 111 sample countries between 1978 and 2017; when defining the historical norm as 30, 40, and 50 years, we use the balanced panel dataset of 147 countries between 1991 and 2017.

### Predicting the potential impact of climate policies on economic growth

**Timing: 5 weeks**

This step selects four climate policy scenarios ranging from stringent to lax, reflecting different scenarios of low to high global average warming by the end of the century. On this basis, the uncertainty impacts of climate damages, policy costs and mitigation burdens on future economic growth are considered by constructing an uncertainty analysis framework.

3. Scenario Settings.
  a. Shared Socioeconomic Pathways (SSPs).
    ***Note:*** We select the SSP pathways that can meet the warming limit targets (e.g., 1.5°C, 2°C, carbon peak and carbon neutrality) in the scenarios provided by CMIP6. For example, the Sustainable Development Pathway (SSP1) and the Fossil-fueled Development Pathway (SSP5).^14^^,^^15^
  b. Representative Concentration Pathways (RCPs).
    ***Note:*** Representative Concentration Pathways (RCPs) refer to an integrated set of concentration and emission scenarios, including a series of radiative forcing different pathways. The RCPs are designed to provide insight into the possible consequences of anthropogenic changes in emission concentrations and associated climate change. They are used as input parameters for climate change prediction models under the influence of human activities in the 21st century.^16^
  c. SSP-RCP scenarios.
    ***Note:*** The CMIP6 combines shared socioeconomic pathways (SSPs) which have socioeconomic implications with representative concentration pathways (RCPs) that reflect GHG emissions, building a common bridge between economic scenarios and climate projections.^17^ This paper mainly considers four representative SSP-RCP scenarios, i.e., SSP1-19 (1.5°C), SSP1-26 (2.0°C), SSP5-34 (global emissions peaking and then achieving net zero or negative emissions) and SSP5-85 (no climate policy).
4. Download future socio-economic forecast data under SSP path.
  a. Log on to the website (https://tntcat.iiasa.ac.at/SspDb/dsd?Action=htmlpage&page=welcome).
    ***Note:*** Download the latest socio-economic forecast data. The SSP database provides country-level baseline economic growth rates for the period 2010 to 2100 under different SSP pathways. The SSP Database provides growth projections generated by three different research groups (OECD, IIASA and PIK); we focus on the projections from the OECD group, as this group predicts more countries than others.

5. Download future climate projections under the four SSP-RCP scenarios.
  a. Log on to the website (https://esgf-node.llnl.gov/search/cmip6/).
    ***Note:*** The CMIP6 produces a series of updated global climate model (GCM) outputs. For our study, we use monthly surface temperature for the future period (2015–2100), and the future temperature projections are provided by dozens of global climate models (GCMs) running under four forcing pathways. Considering the availability of four representative SSP-RCP scenarios, as well as securing the same realization number and initial conditions (variant level “r1i1”), only ten GCMs are available. They are CanESM5, CNRM-ESM2-1, FGOALS-g3, GISS-E2-1-G, GISS-E2-1-H, MIROC-ES2L, IPSL-CM6A-LR, MIROC-ES2L, MRI-ESM2-0 and UKESM1-0-LL. We average these available GCM outputs as future temperature projections. Notably, the horizontal resolutions of the available climate models are different. To get multimodel ensemble mean-based results, we downscale the GCM outputs to a 0.5-degree resolution by using the bi-linear interpolation method.

6. Construction of uncertainty analysis framework.

***Note:*** As we mentioned above, this paper constructs an uncertainty analysis framework with four uncertainty dimensions: (a) climate damages, (b) climate policy, (c) policy costs, and (d) mitigation burdens (see Tang et al. (2023)^1^ for details).

7. Predict future economic growth under climate policies.
  a. Compute the results for each of the 68 scenarios under the uncertainty analysis framework.
    ***Note:*** Based on this framework, this study assesses not only the cumulative impact of climate policies on economic growth across countries (from 2015 to 2100), but also the uncertainty impact of climate policies on future economic growth over different ranges in the near, medium, and long term.
  b. Improve the prediction algorithm.
    ***Note:*** Based on the uncertainty analysis framework, our study further improves the prediction algorithm to more accurately identify the differential policy effects between different RCP scenarios under the same SSP pathway. Specifically, we construct two indices: the “mitigation burden index” and the “moderating damage index” to identify the heterogeneous burden effects of mitigation policies and heterogeneous damages of climate change.

### Calculate inter-country economic growth inequality under climate policies

**Timing: 1 week**

This step measures the degree of inequality in global economic growth for 68 scenarios under the uncertainty analysis framework. Further, the analysis focused on trends in the evolution of inequality dynamics in the median estimates.

8. Measure international economic inequality.
  a. Use 90:10 and 80:20 ratios to calculate international economic inequality.
    ***Note:*** These two ratios use population-weighted country-level empirical cumulative distribution functions (CDFs) of GDP per capita to calculate the degree of difference in GDP per capita between the world’s poorest and richest countries.These two metrics are the eight most popular indices of income inequality identified by Sala-i-Martin.^18^
  b. Calculate the 90:10 ratio.
    ***Note:*** First, the annual GDP per capita of each country is ranked in ascending order for each year from 2015 to 2100; Second, the population of each country is accumulated for each year starting with the country with the lowest GDP per capita. Third, when the accumulated population is 10% (90%) of the total global population, the GDP per capita value of the corresponding country is the 10th (90th) percentile value. Last, the 90th percentile GDP value is divided by the 10th percentile GDP value to obtain the 90:10 ratio.^8^^,^^18^
  c. Calculate the 80:20 ratio.
    ***Note:*** The calculation of the 80:20 ratio is similar to that of the 90:10 ratio, except that the 80th percentile GDP value is divided by the 20th percentile GDP value to obtain the 80:20 ratio. The higher the value of the 80:20 (90:10) ratio, the greater the per capita income gap between the highest quintile (decile) and lowest quintile (decile) countries, indicating a greater degree of inequality in global economic growth.
  d. Calculate the dynamic evolution of global economic growth inequality.
    ***Note:*** Calculate the 90:10 and 80:20 ratios for each of the 68 scenarios in the uncertainty analysis framework between 2015 and 2100 to measure the dynamic evolution of inequality in economic growth.

## Expected outcomes

### Statistical characteristics of the observed climate

Table 1 presents the descriptive statistical results of population-weighted annual average temperature (in °C) and annual total precipitation (in mm) for each country from 1961 to 2017. The global average temperature is generally showing an upward trend. The difference between annual average temperature and historical normal gradually increases, from 0.345°C (twenty years) to 0.573°C (fifty years). During this period, the annual average temperature of each country was 19.985°C, but the climate conditions varied greatly among different countries. In 2010, the average annual temperature in Mauritania was as high as 29.925°C, while in 1979, the average annual temperature in Iceland was only −0.352°C.

**Table 1:** Descriptive statistics of temperature and precipitation from 1961 to 2017

Variable	Mean	Max	Min	Standard Error	Period	Sample
Δlny	0.018	0.319	−0.644	0.048	1961–2017	5016
*T*	19.985	29.925	−0.352	6.804	1961–2017	5016
*P*	12.451	42.998	0.089	6.872	1961–2017	5016
ΔT	0.013	2.599	−2.562	0.506	1962–2017	4928
ΔP	−0.001	16.411	−13.392	2.353	1962–2017	4928
ΔTr	0.061	362.888	−12.748	5.248	1963–2017	4826
deviation *T* (20)	0.345	2.103	0.000	0.292	1980–2017	3344
deviation *P* (20)	1.300	14.330	0.001	1.424	1980–2017	3344
deviation *T* (30)	0.403	2.351	0.000	0.322	1990–2017	2464
deviation *P* (30)	1.358	15.230	0.000	1.487	1990–2017	2464
deviation *T* (40)	0.466	2.471	0.000	0.342	2000–2017	1584
deviation *P* (40)	1.401	15.780	0.001	1.532	2000–2017	1584
deviation *T* (50)	0.573	2.500	0.000	0.402	2010–2017	704
deviation *P* (50)	1.434	11.080	0.002	1.522	2010–2017	704

Note: T (ΔT) and P (ΔP) denote the annual average temperature (change) and total annual precipitation (change) at the national level, respectively. Δlny and ΔTr refer to national economic growth rate and temperature change rate, respectively.

During the same period, Table 1 shows that the difference between the annual total precipitation and the historical climate average gradually increased, from 130 mm (twenty years) to 143.4 mm (fifty years). This means that on a global scale, the overall precipitation has shown an increasing trend over the past 57 years. However, the distribution of precipitation is very uneven and shows significant differences from different sample countries. In 1999, the total annual precipitation of the the Arab Republic of Egypt was only 8.9 mm, reflecting that a serious drought event occurred in the country that year; However, in 1970, the total annual precipitation in Costa Rica was as high as 4299.8 mm, and rainstorm caused huge economic losses.

### Impacts of climate policies on economic growth in different future periods

This study analyzes the economic impact of climate policy from two different temporal perspectives: (1) A cumulative perspective across different periods of this century (2015–2100). (2) Different time scales for the near term, medium term, and long term.

Firstly, we measure the impact of strict climate policies on economic growth in various countries, that is, the relative impact of global warming on economic growth in low RCP scenarios (compared to high RCP scenarios) under the same SSP path.

Then, we analyze the impact of climate policies on the economic growth of 171 countries worldwide from a long-term cumulative perspective of the 21st century (2015–2100). Compared to the 2°C target (SSP1-26), achieving the 1.5°C target (SSP1-19) will increase the per capita GDP of Madagascar, an island country in the African region, by 0.51%. And compared to the high emission scenario (SSP1-26), the probability of Madagascar benefiting under the low emission scenario (SSP1-19) is as high as 71%. Similarly, implementing the 1.5°C climate policy will increase the per capita GDP of Somalia, a coastal country in the African region, by 0.2% with a 54% probability. When looking at poor countries in the Asian region, the per capita GDP of Laos and Cambodia under the 1.5°C scenario will increase by 0.82% and 0.21% with a probability of 100% and 83%, respectively (compared to the 2°C scenario). These results indicate that compared to the 2°C target, the economic benefits of avoiding climate damage under the stricter 1.5°C target may exceed the policy costs. Most of these benefiting countries are the poorest in the world and are concentrated in the climate fragile regions of Africa and Asia.

Furthermore, we explore the economic impacts of climate policies on five major regions (OECD, REF, ASIA, MAF, and LAM) and 18 representative countries from different perspectives in the near, medium, and long term. These 18 countries include nearly half of the world’s population, including the world’s major Economic power, major developing countries, countries prone to natural disasters, and the world’s poorest coastal countries (see Tang et al. (2023)^1^ for details).

According to Table 2, during the Middle century (2024–2055), the median per capita cumulative GDP of the five regions under the SSP1-19 scenario is lower than the median per capita cumulative GDP of each region under the SSP1-26 scenario. Similar results appear in the SSP5 path (SSP5-34 vs. SSP5-85). This means that in the short to medium term, the cost of implementing strict climate policies will outweigh the economic benefits of avoiding climate damage, reflecting that the net benefits of strict climate policies in the short to medium term will be negative.

**Table 2:** Five major global regions: Median predictions of relative changes in cumulative GDP per capita

Middle century (2045-2055)					
	ASIA	LAM	MAF	OECD	Ref.
SSP1-19 vs. SSP1-26	−0.32	−0.65	−1.01	−0.71	−1.62
SSP5-34 vs. SSP5-85	−2.28	−1.65	−2.86	−0.87	−6.03

End of the Century (2090-2100)					
	ASIA	LAM	MAF	OECD	Ref.
SSP1-19 vs. SSP1-26	1.89	1.28	1.58	1.24	2.26
SSP5-34 vs. SSP5-85	2.55	4.47	3.78	5.74	5.25

However, this result will change in the late 21st century. According to Table 2, at the end of the century (2090–2100), the median estimates of cumulative GDP per capita for the five regions under the low RCP scenario (SSP1-19, SSP5-34) exceed the corresponding median estimates for each region under the high RCP scenario (SSP1-26, SSP5-85) in the same SSP.

In terms of the 18 representative countries, as we expected, the stricter the climate policy, the higher the policy cost required at the beginning of its implementation. According to Table 3, implementing the 1.5°C policy will slow down countries’ economic growth in the first half of this century compared to the 2°C policy.

**Table 3:** Eighteen representative countries: Median predictions of relative changes in cumulative GDP per capita

Scenario	SSP1-19 vs. SSP1-26				SSP5-34 vs. SSP5-85			
Period	2030–2040	2050–2060	2070–2080	2090–2100	2030–2040	2050–2060	2070–2080	2090–2100
BGD	−0.92	−0.41	0.52	1.74	−1.86	−2.56	−1.25	2.55
CHN	−1.44	−0.93	−0.04	1.06	−3.05	−3.37	−1.63	3.62
IND	−0.91	−0.25	0.74	1.89	−1.83	−2.36	−1.23	3.21
IDN	−0.81	−0.09	0.97	1.9	−2.2	−3.07	−1.91	2.07
LAO	−0.17	0.83	2.12	3.51	−1.84	−1.99	−0.69	3.39
VNM	−0.88	−0.19	0.81	1.67	−1.72	−1.83	−0.52	3.45
BRA	−0.88	−0.63	0.78	1.35	−1.25	−1.68	0.11	4.76
MEX	−0.80	−0.64	0.82	1.47	−1.27	−1.75	0.16	5.35
IRN	−1.87	−1.18	0.15	1.65	−2.57	−2.88	−0.57	5.13
MDG	−1.06	−0.34	1.01	2.09	−1.78	−2.21	−0.22	3.47
ZAF	−2.62	−2.06	−0.79	0.2	−2.63	−2.9	−0.98	3.53
SOM	−1.36	−0.64	0.59	1.27	−1.96	−1.84	0.29	3.24
GBR	−0.64	−0.56	0.04	0.93	−0.67	−1	−0.05	3.49
JPN	−0.58	−0.34	0.57	1.23	−0.74	−1.16	0.32	4.97
USA	−0.69	−0.57	0.23	0.75	−0.65	−0.81	0.63	5.91
RUS	−3.98	−2.77	−0.76	0.4	−4.2	−6.24	−3.31	5.89
TJK	−2.01	−0.25	1.72	3.73	−3.8	−7.23	−4.04	5.13
UZB	−2.22	−1.03	0.88	2.86	−3.29	−5.95	−2.82	5.69

Besides, we also expect that an immediate implementation of the strict 1.5°C policy would allow vulnerable poor countries prone to natural disasters to reap significant potential gains from the first half of this century. The results of the study are also consistent with our expectations. For example, Laos is one of the least developed countries in the world with poor infrastructure, such as the dam collapse that occurred in 2018, making it difficult to withstand severe extreme weather events.^19^ If global warming continues and exceeds 1.5°C, increasingly severe meteorological hazards such as heavy rainfall and flooding will cause huge economic losses for poor and vulnerable countries like Laos.^20^

### Calculate inter-country economic growth inequality under climate policies

**Figure 5:**
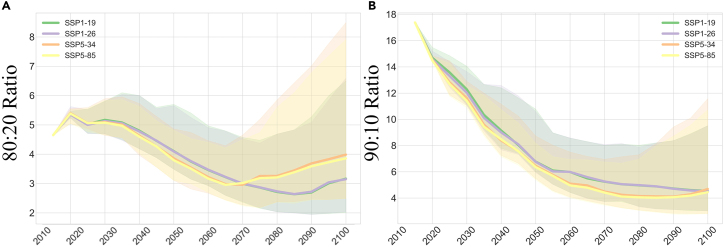
Evolution of economic inequality trends under climate policies (A and B) Note: The dynamic evolution of the inequality index 80:20 ratio (A) and 90:10 ratio (B) between 2015 and 2100, using the equal burden case as an example. The green, purple, orange, and yellow solid lines indicate the estimated median inequality indices for the SSP1-19, SSP1-26, SSP5-34, and SSP5-85 scenarios, respectively. This figure is part of figures from Tang et al. (2023).^1^

We use 80:20 and 90:10 ratios to measure the trend of inequality in global economic growth under climate policies. In this section, we use the equivalent burden case as an example for our analysis. According to Figure 5A, under the SSP5 path, the 80:20 index decreases from 5.40(SSP5-34)/5.38(SSP5-85) in 2020 to 2.92(SSP5-34)/2.94(SSP5-85) in 2065, and then shows a rebounding upward trend and rises to 3.93(SSP5-34)/3.86(SSP5-85) in 2100. A similar situation occurs under the SSP1 path, where the 80:20 index decreases from 5.17(SSP1-19)/5.30(SSP1-26) (2020 year) to 2.59(SSP1-19)/2.61(SSP1-26) (2085 year), and then shows a rebound increase to 3.16(SSP1-19)/3.14(SSP1- 26) (2100 year). These results reflect that, under the same SSP pathway, achieving stricter climate targets for the present would impose a non-negligible economic burden on developing countries later in the century.

Figure 5B illustrates the trend in economic inequality for the 90:10 ratio. In general, economic inequality between the world’s poorest and richest countries shows a decreasing trend. However, in the second half of the 21st century (2050–2100), inequality is slightly higher in the carbon peaking and carbon neutrality scenario (SSP5-34) than in the scenario without any climate action (SSP5-85). For example, the 90:10 ratio in 2100 is 3.93 (SSP5-34)/3.89 (SSP5-85). Again, this result reflects the fact that achieving the double carbon goal will impose an economic burden on the world’s poor countries and requires attention to equitable international burden-sharing mechanisms.

## Limitations

It should be noted that the estimated climate-economic response is calculated from macroeconomic data. Therefore, the differential impact of subdivided economic sectors by climate change is not considered. Besides, economic inequalities caused by cost-sharing policies and income distribution policies are not considered. Therefore, further in-depth studies are needed.

## Troubleshooting

### Problem 1

How to estimate the response between subdivided economic sectors and climate change?

Given the limited availability of sector-level economic output data, this protocol does not assess the impact of climate warming and precipitation anomalies at the subsector level. However, the use of macroeconomic data tends to lose a great deal of useful information, such as the inability to reasonably quantify the extent of climate damage to various economic sectors (related to Steps 1 and 2), which in turn makes it difficult to develop targeted climate mitigation strategies and adaptation measures.

### Potential solution

International trade data are the best available source for identifying global economic activity separately by narrowly defined sectors, and by examining export data, it is possible to more accurately identify which sectors are affected by climate change.^21^^,^^22^

### Problem 2

How to develop an effective and enforceable cost sharing policy?

Given the complex political, economic, historical, and other factors involved in allocating policy costs, this protocol assumes cost allocation based on two typical principles, i.e., equivalent burden and income-related burden, which may not cover all the complicated cases in reality (related to Steps 6).

### Potential solution

Collect more top-down as well as bottom-up cost-sharing data to further explore the development of an equitable international sharing mechanism.

### Problem 3

How to consider the possible uncertainties of temperature and precipitation simulated by CMIP6 models?

For global climate models (GCMs), the biases of temperature and precipitation simulated by different models for different regions are discrepant (related to Steps 5). Therefore, it is important to further perform bias-correction of the simulated temperature and precipitation data in this work.

### Potential solution

Performing bias-correction is necessary if there is only one GCM. In this study, we use the ensemble mean of ten GCMs, which can also reduce the bias caused by a single model. Future research could further perform bias-correction for each model and then average the bias correction for multiple models, thus making our results more robust.

### Problem 4

Socioeconomic development in the long term may increase the resilience of economic growth to climate change.

For example, technological progress may play a positive role in reducing the climate vulnerability of economies, and the estimates in this study may overestimate the extent to which climate damage will affect future economic growth (related to Steps 7).

### Potential solution

Future research could delve into the positive role that technological advances may play in enhancing the adaptability of economies.

### Problem 5

There is a lack of discussion of the impact of climate change on domestic economic inequality.

This study focuses on the impact of climate change on economic growth inequality at the global scale, but does not explore this impact on economic inequality within developing countries. The issue is important because the impact of climate change is more severe in developing countries (related to Steps 8).

### Potential solution

Future research could delve into the impact of climate change on economic inequality within developing countries. For example, explore the impact of climate change on domestic economic inequality in the Chinese context, and further explore the differential impact of climate warming on China and the world.

## Resource availability

### Lead contact

Further information and requests should be directed to the lead author, Hongbo Duan (hbduan@ucas.ac.cn).

### Materials availability

This study did not generate new unique materials.

### Data and code availability

The sources of the data sets supporting the current study have been presented. The other data and codes can be available on request from the lead contact.
